# To Spray or Not to Spray: A Decision Analysis of Coffee Berry Borer in Hawaii

**DOI:** 10.3390/insects8040116

**Published:** 2017-10-21

**Authors:** A. John Woodill, Stuart T. Nakamoto, Andrea M. Kawabata, PingSun Leung

**Affiliations:** 1 Department of Natural Resources and Environmental Management, College of Tropical Agriculture and Human Resources (CTAHR), University of Hawaii at Manoa, Honolulu, HI 96822, USA; psleung@hawaii.edu; 2 Department of Economics, College of Arts and Sciences, University of Hawaii at Manoa, Honolulu 96822, HI, USA; 3 Department of Human Nutrition, Food and Animal Sciences, CTAHR, University of Hawaii at Manoa, Honolulu, HI 96822, USA; snakamo@hawaii.edu; 4 Department of Tropical Plant and Soil Sciences, CTAHR, University of Hawaii at Manoa, Honolulu, HI 96822, USA; andreak@hawaii.edu

**Keywords:** coffee berry borer, *Hypothenemus hampei*, integrated pest management, decision analysis, pest control

## Abstract

Integrated pest management strategies were adopted to combat the coffee berry borer (CBB) after its arrival in Hawaii in 2010. A decision tree framework is used to model the CBB integrated pest management recommendations, for potential use by growers and to assist in developing and evaluating management strategies and policies. The model focuses on pesticide spraying (spray/no spray) as the most significant pest management decision within each period over the entire crop season. The main result from the analysis suggests the most important parameter to maximize net benefit is to ensure a low initial infestation level. A second result looks at the impact of a subsidy for the cost of pesticides and shows a typical farmer receives a positive net benefit of $947.17. Sensitivity analysis of parameters checks the robustness of the model and further confirms the importance of a low initial infestation level vis-a-vis any level of subsidy. The use of a decision tree is shown to be an effective method for understanding integrated pest management strategies and solutions.

## 1. Introduction

The coffee berry borer (CBB), *Hypothenemus hampei* (Ferreri), is one of the most destructive pests of coffee, second only to coffee leaf rust in the damage it causes. Hawaii was one of the last coffee producing regions not infested; that is, until 2010 when the first signs of CBB’s arrival to the islands were documented in Kona, Hawaii [1]. Some farmers reported infestation levels of up to 80–90% of their crop, whereas processors currently reject a crop with more than 25% damage. Bearing acreage is down 16% in the last three years and in the most recent 2015–2016 season , over 2.6 million lbs. of cherry (fruit containing the coffee bean) were rejected by processors. The value of utilized production is also down from $62 million in 2014–2015 to $49 million in 2015–2016 [2]. Even before the arrival of CBB, many farms operated on small margins [3] with costs and uncertainty driving the shutdown of farms. Poorly managed or abandoned farms and feral coffee provide reservoirs for CBB, thus further contributing to the problem.

CBB is prevalent throughout the coffee producing regions of the world but may not be as big of a problem due to, among other reasons, the use of toxic chemicals. Because of the toxicity of these chemicals, they are banned in the United States so other methods for dealing with CBB must be used. Integrated pest management (IPM) strategies were adapted from elsewhere to aid farmers in responding to the pest with recommendations on monitoring, spraying, harvesting, and disposal [4]. While benefits to spraying exist, the effectiveness is limited, pesticides and spray labor are costly, and much is unknown about the application rates, efficacy, the effect of weather and environmental conditions, and other farm-level components. Further, production areas are not uniform due to differences in elevation and terrain, farm size characteristics, management styles, and growers’ management philosophies. The lack of information on these issues and the significant damages caused by CBB warrant the need for an economic study of decisions being made on a farm.

Previous research by Leach and Stonehouse [5] focus on coffee cherry production and CBB population dynamics in the Caldas region of Colombia and cost-benefit harvesting strategies, but suggest their results are exploratory at best. A 3-year study of IPM strategies in Colombia showed the methods were effective in reducing infestation with proper management and training programs [6]. Further, strategies developed in Latin America provide recommendations for Hawaii farmers, extension technicians, and research centers to combat the impact of CBB [7]. Other research in Hawaii has studied traps and lure formulations with results that suggest many challenges ahead for the management of CBB in Hawaii [8]. The goal of this paper is to incorporate a decision tree framework as an exploratory analysis of the impact of CBB on a farmer’s net benefit and advance baseline knowledge for farmers, researchers, and policymakers in Hawaii. We intend to add new insights into the economic impacts of CBB in Hawaii and offer a decision analysis to combat these effects.

A natural starting point for modeling decisions, and the one used in this paper, is a decision tree analysis where the individual decisions in each month of a growing season are optimized. Decision trees are simple to use in being able to model economic farm-level pesticide use decisions with the data we have available, but powerful enough to answer farm-level decisions. Further, farmers are able to use the model for their own farms and visualize the decisions with familiar software (Microsoft Excel). Thus, the decision tree is a perfect fit for our modeling approach. For a detailed review of decision analysis methods see [9,10,11,12,13,14].

In the context of our paper, a decision tree provides a visual representation of the decision making process for a farm by mapping a wide variety of usually sequential decisions in order to determine an optimal decision path. A farmer chooses to either spray/not spray at each period (month) for the entire growing season (year). Inputs for parameters on the farm (acreage, coffee cherry yield per acre, etc.), pesticide spraying information, CBB growth rates and infestation levels. With CBB, there are various stages (i.e., A, B, C, D) of entry into the bean where spray is effective or not. In this model, “infestation level” is used as a proxy for all stages, but the end result is the percentage of the harvest that is damaged and therefore not marketable, i.e., CBB are in the C/D position [4]. Coffee harvest rates are evaluated to determine the net benefits of the possible decisions in each period, which in turn are summed over the season. The entire map of decisions shows each period (node) and highlights the optimal path that maximizes the total net benefit. This provides an advantage in understanding sequential uncertainty in each period and also a visual representation of the decisions a farmer makes.

Our approach follows the conventional framework for a decision tree, where firms seek to maximize their expected utility (benefit) at each node, which lays out a map of decisions over a specified time period [15,16,17]. Decision analysis has been applied extensively in business and manufacturing (for a review see [18,19]), in agriculture with dairy cow hormone decisions [20], in forestry with applications to uncertainty and optimal decision solutions to deal with gypsy moths [21], and in pest control [22]. The authors focus on a cost-benefit analysis to identify strategies for optimal net benefit. Empirical estimations using spreadsheet modeling is outlined in Ragsdale et al. [23] and is used as a basis for our modeling approach.

Farm-level knowledge and management practices play an important role in understanding and dealing with risk and uncertainty in agriculture [13]. Risk and uncertainty can come from various areas on a farm, such as the inability of a farmer to accurately account for the infestation level in a period [24], the population dynamics (density-impact curve) of the pest [25], and the risks of the actual decision itself by the farmer [22]. Improved information can overcome these issues and help mitigate risk and uncertainty, which ultimately reduces costs from misinformation or imperfect knowledge. Another study further shows that collaboration of information between theory and empirics is important in dealing with decisions under uncertainty [26]. Due to the recent discovery of CBB, data limitations, and research in progress, understanding the uncertainty of CBB population dynamics is problematic in Hawaii. To address this limitation and help reduce the uncertainty, we have calibrated the infestation level and rate in our model based on farm-level knowledge and field testing of a typical farm in Hawaii. We further account for uncertainty by testing the robustness of our results using sensitivity and breakeven analyses of parameters established in the model.

Another important element of decision tree analysis in pest management is economic threshold, which is defined as the infestation level where it is beneficial to control (spray/not spray) a pest given the potential for economic damage [27,28]. Economic threshold determines the tradeoff between the costs of spraying compared to the economic damages that are avoided. The threshold defines this tradeoff as an inequality of the cost to control as less than the difference between price times yield and price times damages [29]. As long as the cost to control is less than the economic damage resulting from no control then it is beneficial to spray. We use economic threshold in our decision tree model to understand the decision made in each period and whether it is beneficial to spray or not spray based on the damages from CBB and costs associated with spraying.

This paper contributes to the literature in three ways. First, we use a decision tree framework to quantify the bridge from theoretical to an empirical application by using the economic threshold to guide the decisions made by a farmer. All possible pest management decisions for a coffee farm in Hawaii are mapped to determine the optimal path that maximizes net benefit for a farm during a growing season. We rely on ongoing farm-level research to provide data for accurate model calibration.

Second, simulations, sensitivity, and breakeven analyses provide insight into the impacts of parameters and their significance on farm-level decisions. This also allows us to reduce uncertainty in the model by providing accurate farm-level knowledge and tests on the sensitivity of the parameters. Due to the current lack of concrete data, sensitivity analysis is provided in lieu of methods of stochastic variability.

And third, two main results suggest the initial infestation level in a crop season is the most important component of farm-level decisions and while a subsidy does provide a benefit to farmers, it does not mitigate damages done from not ensuring a low initial infestation level. These contributions provide a tool to help farmers make pest management decisions, to provide researchers with insights into their work, and to guide recommendations and policy making.

## 2. Theoretical Optimization Model

The primary action in our decision tree model is whether the farmer decides to spray or not spray a pesticide in each period—or each month in a 12 month growing season—based on the trade-off between damage from infestation and cost to spray as the economic threshold. As described below, we rely on modeling the infestation level and harvest rate to optimize the net benefit over the entire growing season.

### 2.1. Infestation Level

The first component of the model addresses the CBB infestation level and the rate at which that level increases on a farm. Generally, this is an entomology problem because the growth rate of a pest is based on the biological development as it relates to various environmental conditions. Understanding the growth rate is only in the early stage of research for CBB in Hawaii, so incorporating an entomology infestation model into decision theory is not possible. To overcome this limitation an estimate for each infestation level at each period based on the growth rate of CBB infestation on the farm is incorporated into the model.

We define the infestation level, $I_{t}$, as a percentage of the total harvest of coffee cherry that is damaged and not marketable in accordance with Hawaii grading standards. Once a cherry is infested (damaged) it cannot be reversed, thus the infestation level cannot go down. The only way to reduce the infestation level increase is to spray the farm, which comes at a cost. The equation for the infestation level considers whether the farm was sprayed or not:

(1) $$I_{t}=I_{t-1}+G_{t}\cdot I_{t-1}\left(1-E_{t}\right)$$

Equation (1) defines the infestation level, $I_{t}$, given the choice to spray or not spray at time period *t* based on the previous infestation level $I_{t}$ times the growth rate, $G_{t}$. The growth rate is defined as the percentage increase in the infestation level. When a farmer decides to spray the infestation level changes based on the effectiveness rate, $E_{t}$, of the pesticide, thus reducing the growth rate of CBB infestation in period *t*. The effectiveness rate is the percentage decrease in the growth rate due to spraying, thus reducing the level of damage to the coffee bean. CBB have not shown resistance to the main pesticide spray, *Beauveria bassiana*, in Hawaii, so we assume effectiveness does not decrease with additional spraying. If a farmer decides not to spray, then the effectiveness equals zero and the infestation level increases by the growth rate of infestation. This infestation level is then carried out through each period at each decision (spray or not spray) based on the growth rate of CBB infestation.

It is possible for the infestation level to increase rapidly based on decisions in previous periods. For example, suppose a 20% infestation level at time period $t_{1}$, a 50% spray effectiveness, and a 35% growth rate. Spray effectiveness is defined as the kill rate from spraying a pesticide. A 50% spray effectiveness assumes 50% of the current population will die from spraying. This definition can also be described as the marginal spray effectiveness, but we use spray effectiveness throughout the paper. If a farmer chooses to not spray their infestation level in time period *t* will be 27% and if they choose to not spray again this will increase in the next period t+1 to 36.5%, with a doubling of the original infestation (and associated crop damage) within three periods. However, if a farmer chooses to spray in each period the first period infestation level will be 23.5% and the next period 27.6%, with a doubling within five periods. Farm-level decisions ultimately play a large role in the infestation level. Decisions of neighboring farms may also play a role in the infestation levels, but the effects are currently not well understood in Hawaii. We focus on individual farm-level decisions to asses how infestation levels change and neglect potential spill-overs from spraying or not spraying by a neighbor.

### 2.2. Harvest Rate

Many factors come into play as a farmer decides to harvest, but for the purpose of this model a farmer projects a specific percentage of their total crop to be harvested in each period. Each period’s harvest is affected by the infestation level that lowers the net benefit in that period. Net benefits are then summed across all periods with the total net benefit calculated in the final period. For example, suppose a farmer decides to harvest 25% of their crop in each of the final four months. There is a higher net harvest in the first month of harvest because of a lower infestation level. With an increasing infestation level, subsequent net harvests are lower regardless of spraying or not, with the lowest net harvest in the final month. A simple optimization strategy would be to harvest your entire crop in the month with the lowest infestation level. However, this is not the case when harvesting coffee because the cherry does not ripen all at once, but is instead spread out. In any given harvest period the following equations define the net harvest. Summing across all harvest periods *T* provides the total net harvest.

(2) $${\mathrm{Harvest}}_{t}=A\times J\times H_{t}\left(1-I_{t}\right)$$

(3) $$\text{Total Harvest}=\sum_{t=1}^{T}A\times J\times H_{t}\times \left(1-I_{t}\right)$$

Equation (2) defines the net harvest in time period *t* as the cherry harvest minus cherry damage—no price for cherry or costs associated with production are included in this equation. Equation (3) defines the total net harvest as the sum of net harvests over *T* periods where *A* is the total acres on the farm, *J* is the projected pounds of cherry per acre, $H_{t}$ is the percentage of the total harvest in period *t*, and $I_{t}$ is the infestation level at the harvest period *t*.

### 2.3. Net Benefit

The net benefit (NB) takes into account the projected revenue from the net harvest of coffee cherry, and costs associated with spraying and harvesting. The following equations outline the net benefit in each period given costs and is then maximized across all periods in the season to arrive at the total net benefit.

(4) $$NB_{t}={\mathrm{Harvest}}_{t}\times P-C_{t}$$

(5) $$C_{t}=A\times \left(HC_{t}+SC_{t}\right)$$

(6) $$\max NB=\sum_{t=1}^{T}NB_{t}$$

Equation (4) defines the net benefit for a given period where NB is net benefit, ${\mathrm{Harvest}}_{t}$ is from Equation (2), *P* is the price of cherry and is exogenous and assumed constant. The price is associated with cherry not damaged by CBB (marketable). Costs, $C_{t}$, associated with spraying and harvesting (e.g., labor, chemical costs, water use, and equipment) are defined in Equation (5) where $HC_{t}$ are the harvest costs at time period *t* times farm acres, *A*, plus spray costs ,$SC_{t}$, at time period *t* times acres, *A*. Harvest costs include cherry that is damaged because when harvesting cherry the farmer does not inspect each individual cherry—this is normally done by the buyer at time of processing. Equation (6) defines the objective function by maximizing the total net benefit where all periods are summed to arrive at the projected benefit a farmer receives from their entire crop. In each period (i.e., at every decision, spray or not spray) a net benefit is calculated and then summed across all *T* periods. If a farmer decides to not spray then they will not incur costs, but their infestation level will rise more than if spraying, thus lowering the net harvest and ultimately lowering their total net benefit. A farmer who sprays incurs costs, which lowers their total net benefit, but the reduction in infestation level improves the harvest benefit. The tradeoff between spray cost and value of crop saved by spraying determines whether the farmer would spray or not spray in each period.

The infestation level, net harvest, and net benefit interact together to provide a model able to describe a particular farm and the decisions made by the farmer based on various inputs and conditions on the farm. The infestation level ultimately drives net harvest and total net benefit, but the farm-level decisions determine the infestation level. Careful consideration of farm-level decisions is necessary to ensure the model accurately describes a farm and the total net benefit as a result of those decisions.

## 3. Empirical Decision Tree Model

To optimize the net benefit, a decision tree models each decision as a node in the tree. Each node takes into account the infestation level, harvest, and decisions at that period in time and calculates the optimization parameters. The software we use is Risk Solver Platform^®^ [30] (Frontline Systems Inc., Incline Village, NV, USA), which is a Microsoft Excel Add-In. This software was chosen for its ease of use and familiarity for farmers. The parameters for a farm are assigned to cells used in a spreadsheet, and the decision tree takes each node and applies the equations for infestation level, harvest, and net benefit. Three separate trees are mapped out as discussed by the theoretical equations.

Table 1 outlines the variables used, the associated units, possible ranges, and an estimated average for a coffee farm in Kona, Hawaii. Table 2 outlines the variables related to the infestation level. These variables are not necessarily related to farm-level decisions but instead provide information necessary to calculate the infestation level.

Calculating the infestation level of CBB in Hawaii is dependent upon many factors, such as weather conditions and elevation changes, and current estimates are still in the early stages of research. To overcome the problem of a lack of data to accurately model the growth rate, we use data from field tests on coffee farms in Hawaii to calibrate the infestation levels and growth rate [31,32]. The field tests were conducted using various spray applications under spray/no spray conditions to understand the changes in infestation using micro plots. The micro plots were randomly sampled each month, and in the samples ripe “cherries” (coffee fruit) and infested green fruit were removed to assess the state of infestation and calculate totals. We use these results to calibrate infestation levels under different spray patterns.

To calibrate the infestation growth rate we assume a typical farm with an initial infestation of 1% and a spray effectiveness of 50% (see Table 2). We use the infestation level data at the end of the season for plots that were always sprayed and never sprayed. The plot that was always sprayed could expect a final infestation of around 6% and when never sprayed a final infestation of 25%. A constant infestation growth rate of 35% accurately reflects the field-level data and was chosen as the best fit overall for the model.

We assume throughout the model that integrated pest management recommendations are followed as outlined in Kawabata et al. [4]. Specifically, proper field sanitation practices are followed (e.g., stripping remaining fruit from trees at the end of the season) to maintain lower infestation levels, farmers monitor their fields to determine current infestation levels each month, decide to spray once per month, and harvest available cherry on the farm if available in the current period. While we don’t explicitly model each of these recommendations, we imply they are being followed. For example, when testing low initial infestation levels vs. higher initial infestation levels we assume the farm with lower levels followed the field sanitation recommendations better than the farm with higher initial levels. This allows us to test likely outcomes from the recommendations and determine their relevance.

The decision tree takes into account 6 periods—each period is 2 months due to limitations of the software—for a total of 12 months, or the entire growing season. At each period (2 months) two decisions exist: (1) spray; or (2) not spray. The decision tree is configured in a way to allow for each month to account for individual parameters, but the decision to spray or not is made on a per period basis. Each decision depends on past decisions, so the tree accounts for all possible decision paths. The final period contains a total of 64 nodes that trace every possible combination of spray decisions in a growing season.

The model starts with the infestation level tree to account for changes in the CBB level through time. Starting from the initial infestation level, in each period the growth rate establishes how much a farm can expect CBB infestation to increase if there was no spraying in the period, while the spray effectiveness decides how much a farm can reduce the infestation growth rate by spraying. Damaged coffee beans cannot be healed, so an irreversible infestation level is assumed with no negative growth rate. It is beneficial to spray at each period in order to minimize the infestation level, which may not be the optimal decision because the cost of spraying is not yet included. The infestation level tree accounts for damages from CBB in each period and is used to determine the harvest benefit.

We use a harvest tree to account for cherry on the farm that is ripe and ready to harvest in each period. As discussed above, cherry do not ripen all at once, but generally is spread out over the last four months of the season. Further, the cherries that ripen in the later months will have higher levels of infestation due to being exposed to CBB for a longer period, or due to CBB being disrupted when cherry is harvested and relocating to other cherry. As a simplification, we assume 25% of the projected crop is harvested in each of the last four months of the season. The amount of cherry harvested is removed from the projected cherry total, damaged cherry due to infestation are removed and considered non-marketable, and the remaining cherry is priced at the market value of $2. Controlling for harvesting in this way allows the model to account for non-uniformity in cherry ripeness and the impact of different infestation levels at different periods.

Finally, the net benefit tree ties everything together. First, for each node, a price for the cherry is applied to the net harvest and the costs of spraying and harvesting are subtracted to arrive at the net benefit. The net benefits for the nodes are summed across the growing season to determine the total net benefit for each path. By comparing total net benefits, the farm can derive the optimal decision strategy based on the objective of maximizing its total net benefit.

## 4. Results

### 4.1. Simulation Study 1: Minimizing Initial Infestation

In general, if a farmer implements farm-level sanitation efforts they will start the season with a lower initial CBB infestation level. As the level of sanitation decreases, the initial infestation level increases because more CBB are left from the previous season. These initial conditions can have an impact on how a farmer manages their infestation levels throughout the year and ultimately impacts their final net benefit. Thus, the first simulation study asks: “Is it beneficial for a farmer to keep their initial infestation level low by taking care of farm sanitation and preventative measures for CBB?”

**Figure 1:**
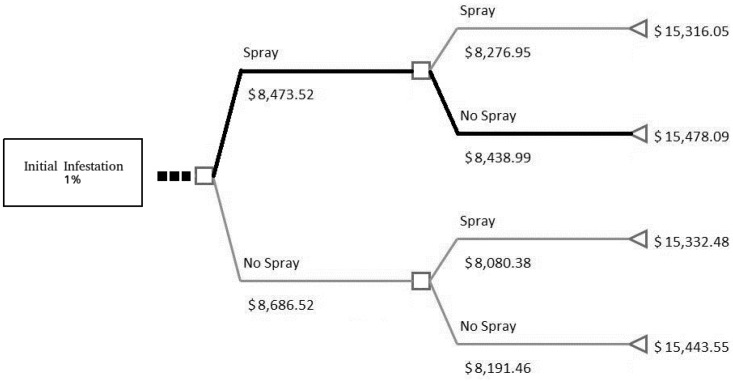
Scenario 1 with 1% initial infestation, last two periods: this figure is a close-up of a decision tree and the final two periods based on scenario 1 in the text. The squares represent a decision to spray (**up**) and not spray (**down**). The numbers below represent the net benefit for that period based on the decision. The left triangle is the end point, or final month of December, with the number representing the total net benefit. The black line represents the optimal decision path.

**Figure 2:**
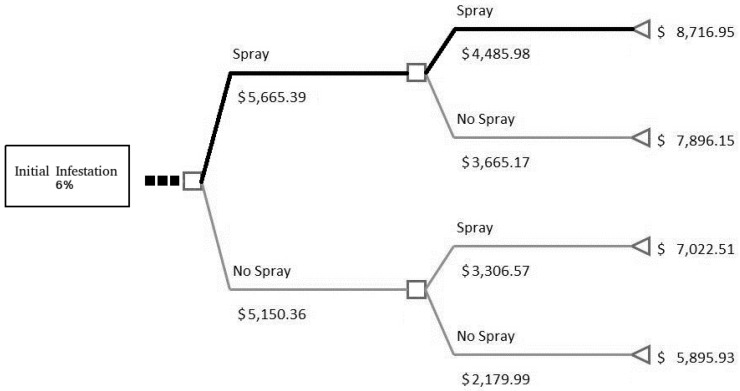
Scenario 2 with 6% initial infestation, last two periods: this figure is a close-up of a decision tree and the final two periods based on scenario 2 in the text. The squares represent a decision to spray (**up**) and not spray (**down**). The numbers below represent the net benefit for that period based on the decision. The left triangle is the end point, or final month of December, with the number representing the total net benefit. The black line represents the optimal decision path.

To answer this question, parameters used to describe a typical farm and the initial infestation level are tested using 1% and 6% (scenarios 1 and 2). In scenario 1 (Figure 1) we assume the farmer took the necessary steps to ensure a lower initial infestation level than in scenario 2 (Figure 2). Both figures show the last two periods (4 months) and the net benefit for each period—see net benefit in dollars under spray/no spray—followed by the total net benefits for each decision path at the far right. In this case, each of the past decisions are to always spray up to the ellipses in the figures with divergence in decisions outlined by the thick black line. The assumptions necessary for these two scenarios are a: (1) 35% constant growth rate; (2) 50% spray effectiveness, and (3) constant costs of $179.30 per month in which spraying occurs.

The results of this analysis demonstrate it is beneficial to keep the initial infestation level low by taking the necessary steps early rather than later. Table 3 provides the total net benefit and illustrates the differences between the scenarios are quite large. The difference is a positive net benefit of $6761.14 for an initial infestation rate of 1% instead of 6%. Therefore, as long as the cost in the beginning of the season to keep the initial infestation rate below 1% is less than this difference, it is beneficial to attain a lower initial infestation level. By achieving this, a farmer is able to obtain a higher net benefit in a growing season. As shown in the sensitivity analysis below, the initial infestation level is the most important factor controllable by the grower at the farm-level.

The costs associated with maintaining a low initial infestation level relate to the efforts needed to sanitize a farm during the end of the current season and the beginning of the succeeding season. The main efforts include picking up coffee off the ground, strip picking berries from the trees, and the use of stumping as a method of pruning [4]. Picking up cherry from the ground can be the most labor intensive due to the rocky terrain and uneven landscape. We estimate the time to pick up cherry from the ground to be between 25–35 hours per acre at a cost of $375–$525 ($15/hour). Strip picking takes 15–25 hours per acre depending if the farmer chooses to strip pick only at the end of the season, or again just before the next season begins, so a farmer would incur a cost of $225–$375 per acre. Stumping/pruning is a necessary effort even without CBB, so we assume zero cost to decrease infestation levels. As our simulation exercise suggests, as long as the costs to sanitize a coffee farm is less than the difference between infestation levels ($6761.14 in the simulation above) then it is beneficial to sanitize. At an estimated cost of $1002–$1503 for a 1.67 acre farm it is almost always beneficial to sanitize the farm prior to the season in order to maintain a low initial infestation level.

As an added bonus, Figure 1 shows that with a low initial infestation level, it is not always necessary to spray in the final period, thus increasing the final net benefit over always spraying by $162.04. This, however, is not always the case as the difference between spraying and not spraying is relatively small, and is seen as an added benefit to this case study.

### 4.2. Simulation Study 2: Impact of Subsidy

The Hawaii Department of Agriculture provides a subsidy for the pesticide to combat CBB. It is useful to understand the implications of the lower product cost of the farmer’s spray decisions, as well as likely impacts when the subsidies eventually end.

Two scenarios were applied to the decision tree model to test the impact of this subsidy as it is now and the effects when it ends. First, all average conditions from Table 1 were included to model the final net benefit when there is no subsidy. Then, a second scenario was incorporated with a subsidy. Currently, farmers are able to buy the subsidized spray at $15 per quart, which is the recommended application per acre. The results of this analysis show the subsidy is currently providing an additional $947.17 net benefit under the optimal management strategy with cost savings of $92.43 per month for a farm with 1.67 acres (Table 4). Additionally, because of the low cost of the subsidy, the optimal decision under the subsidy is to spray in every period – including the last period. If farmers follow the optimal strategy of not spraying in the last period as in Scenario 1, net benefits are reduced by $22.83 (see last row in Table 4).

While the subsidy provides a benefit to farmers from a reduction in costs, it is important to note the difference between the benefit provided under the subsidy and the results of the initial infestation level described in the first simulation study. A subsidy to a farmer reduces costs and increases the final net benefit at the end of the season; however, this subsidy does not mitigate damages done to a crop by not starting with a low initial infestation level. If a low initial infestation level is not maintained in the beginning of the season then the final level of CBB infestation will be much higher, thus removing any benefit provided under the subsidy. If, however, necessary measures are made at the farm-level to ensure a low initial infestation level, then the benefits of the subsidy will be realized by a reduction in spray costs. This paper shows the benefit of the subsidy only exists in conjunction with a low initial infestation level.

## 5. Robustness Checks

The uncertainty of parameters in the decision tree model can significantly change the total net benefit. This uncertainty stems from unknown farm-level knowledge or the uncertainty of knowing whether those parameters are accurate, as well as the effects of external factors such as the weather. Sensitivity analysis allows for a better understanding of the relationship between input variables and how the total net benefit and optimum decision path are impacted. Furthermore, sensitivity analysis allows for checks on the robustness of assumptions and parameters over a range of estimates, thus providing a confirmation of the model calibration. Breakeven analysis offers another useful tool by looking at the specific level of each parameter given a zero net benefit condition. This further confirms the sensitivity of parameters and offers further robustness checks on the interaction effects.

### 5.1. Sensitivity Analysis

To check the robustness of the model, a typical farm in Kona is used as the basis for the sensitivity analysis. A typical farm has 1.67 acres of coffee and an estimated yield of 7500 lbs. of cherry per acre. Five parameters are changed based on a range and base (See Table 5). The min and max for each parameter provide the ranges within the sensitivity analysis and the base provides the assumed baseline values. Two scenarios are included in Table 5 that provide pessimistic and optimistic parameter values.

To illustrate the need for sensitivity analysis, a simple exercise is provided to get a sense of how specific conditions may change the net benefit and spray strategies. As an example under pessimistic conditions, assume a base spray cost of $70.35, a cherry price of $1.50 per pound, spray effectiveness of 40%, and a higher growth rate and initial infestation level of 50% and 4%, respectively. Under these conditions, the final net benefit is $2977.12 with a spray strategy of always spraying in order to maximize the final net benefit. Assuming a more optimistic condition when the farmer gets the full price of $2 per pound of cherry, spray costs are at the base rate of $70.35, spray effectiveness of 50%, and a lower growth rate and infestation level of 25% and 1%, respectively. Under these conditions, the final net benefit increases to $16,447.40 with a spray strategy to spray in the first ten months, and then do not spray for the remaining two months of the season. The two different scenarios provide different final net benefits and spray strategies. Thus, testing specific scenarios and determining the sensitivity of the parameters under certain conditions help provide more information about decisions on a farm.

**Figure 3:**
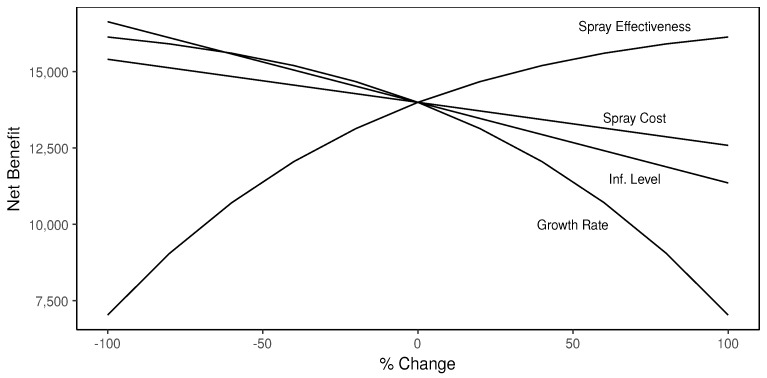
Spider Plot Showing Percentage Change in Net Benefit: This figure shows the change in net benefit as spray effectiveness, spray cost, infestation level, and growth rate changes from +/− 100%.

The results from simulation study 1 suggest the final net benefit is sensitive to the initial infestation level. Figure 3 provides a visual representation in the form of a spider plot to understand how a percentage change in one of the parameters listed above impacts the net benefit. These results confirm the initial infestation has a greater impact on net benefit than spray costs. The growth rate of CBB infestation and spray effectiveness of the pesticide have significant changes to net benefit and share a nonlinear effect possibly due to the compounding effect of CBB infestation rate per period. As spray effectiveness increases or the growth rate of infestation decreases there is less of an impact on the final net benefit.

The initial infestation is not realistically reflected in Figure 3 because a 100% (+/−) percentage change from 1% initial infestation is only 0% or 2%. As mentioned before in simulation study 1, this is not realistic because the infestation is likely to be much higher (e.g., 6%) if certain precautions are not taken. Table 6 looks at the sensitivity of the final net benefit to the initial infestation level. As shown, the initial infestation level has a major impact on the net benefit; whereby the difference between 0% and 5% is $6946.42. Another interesting result is any level of initial infestation above 12.6% (breakeven) will produce a negative net benefit, which suggests if the initial infestation level is too high then treatment does not provide any benefit since spraying cannot kill enough CBB to improve the net benefit. This suggests the final net benefit is sensitive to the initial infestation level and further confirms the main result about the importance of the initial infestation level.

The spray effectiveness sensitivity analysis is provided in Table 7. The results suggest the spray effectiveness is also an important factor in the final net benefit, but may not have as large of an impact as the initial infestation level (see differences in net benefit in Table 6 vs. Table 7). As shown, the impact of a change in the spray effectiveness from 0 to 100% is $2792.28 for net benefit and 2114 lbs. of cherry for net harvest. There is a much larger effect on the final infestation level with the difference of 21.1%. Another interesting point to note is the spray strategy changes throughout the season. As might be expected, with a spray effectiveness of 0% a farmer never sprays.

If the spray effectiveness is at 25%, then it is beneficial to spray only the first 6 months, and when effectiveness is at 50%, then only spray up until the final period. And finally, if spray effectiveness is at 75% or 100%, then only spray up until the last 4 months, or potentially when harvesting occurs.

The sensitivity check of optimal net benefit to the pesticide cost shows that with a full subsidy the added benefit is $1247.77 (Table 8), which is a difference of $300.60 in total net benefit compared to the current subsidy rate of $15. The difference between having an initial infestation level of 0% and 1% is $1389.28 (Table 6), which is larger than having the benefit of a full subsidy $1247.77. This further confirms the value of a lower initial infestation level; in comparison, pesticide costs are not as impactful on the optimal net benefit. No matter what the reduction in the cost of the pesticide through a subsidy, it is more important to start with a low initial infestation level in order to maximize the final net benefit.

### 5.2. Breakeven Analysis

A breakeven analysis for a parameter identifies the value where net benefit is zero. Table 9 provides a breakeven analysis for the initial infestation level when spray cost and spray effectiveness are set at selected levels. For example, when the spray cost is at $50, the breakeven, or zero net benefit, occurs when the initial infestation level is at 12.91%. Or, if spray effectiveness is at 50%, then the breakeven occurs when the initial infestation level is at 12.1%. An interesting result of this analysis shows that spray cost has a minimal impact on the breakeven. Note the range of pesticide costs are $0-$100, but the initial infestation level is only between 13.67–12.15%. This suggests there is only about a 1.5% difference in the breakeven between a cost of $0 and $100. The breakeven analysis suggests that with any initial infestation level, the spray cost will not have much impact on final net benefit. Net benefit is insensitive to spray cost and reinforces prior results.

Again, spray effectiveness appears to have a large positive impact on the net benefit—also confirmed due to the non-linear effect shown in the spider plot. The spread is much larger for the breakeven as it relates to the initial infestation level. At a spray effectiveness of 0%—where the growth rate of CBB for spraying and not spraying are equal—the initial infestation level must be at 3.47% to breakeven. On the other end, if the spray effectiveness is at 100%—or no growth of CBB infestation in each period—then an initial infestation level of 66.41% can occur for a farmer to breakeven. Thus, farm profitability will depend on the combination of spray effectiveness and initial infestation level. With an ineffective pesticide, the initial infestation level must be low to get a positive net benefit. If a more powerful pesticide is used the farmer will have greater control so a positive net benefit still exists even if the initial infestation level is much higher. We do not currently have data on the costs associated with obtaining different levels of initial infestation so the net benefit would likely still be maximized at a lower initial infestation level.

As a final robustness check, the base case is modeled to find the net benefit breakeven levels for pesticide cost, initial infestation level, spray effectiveness, growth rate, and price assuming a single spray in each month (Table 10). Assuming all other parameters are set to the base (See Table 1 and Table 2), the pesticide cost needs to equal $834.62 in order for a farmer to return a zero net benefit. Furthermore, spray effectiveness would be negative 59.29%—suggesting that spray effectiveness is harmful and increases the number of CBB; growth rate increases to over 112%, and the price drops to $0.71 in order for the farmer to breakeven. These numbers are improbable, suggesting that as long as a farmer undertakes monthly spraying throughout a growing season at every month then they will turn at least a positive net benefit. However, given the base conditions, the initial infestation level needs to only be 12.6%, above which net benefits are negative. This number is easily reached and surpassed if certain precautions were not taken into account, such as cleaning the farm, or strip picking. The results of this analysis confirm that the initial infestation level is again the most important factor as it relates to the final net benefit.

## 6. Discussion

Our decision tree model is effective in testing and understanding integrated pest management solutions. Results of the model, verified by sensitivity analyses, show two significant findings. First, at the farm level, the initial infestation level is the most important factor to maximize the final net benefit. An increase in the initial level from 1 to 5% reduced the net benefit by $5000. Higher spray effectiveness and lower pesticide costs only increased net benefits slightly. A breakeven analysis further shows a zero net benefit results at 12.6% initial infestation.

The first finding is significant because it suggests pre- and early season preparation is a critical factor for keeping infestation levels low throughout the growing season. A farm that starts off with high levels of infestation can never reach crop damage levels below a farm with a low initial infestation level. Even with the ability to spray every month, if the farm is not cleaned and prepared for the season then no amount of spraying can reverse the damage.

Second, these results hold when there is a subsidy. We estimate that the pesticide subsidy program provides an annual positive net benefit per farm of $947. With low initial infestation levels, the subsidy is able to further reduce spraying costs and helps to mitigate damage to the crop. More importantly, no subsidy level and associated amount of spraying will undo damage done by not ensuring a low initial infestation level. This is also significant because the subsidy is only legislated for a short period of time, so will not be available to offset costs indefinitely.

From a policy perspective, our results suggest that instead of subsidizing the cost to spray, it may be more important to subsidize the cost of field sanitation. This policy recommendation aims for farmers to not only rely on spraying to reduce infestation levels, but also to take action to maximize the benefits from spraying, that is by starting the season with lower levels of initial infestation. Otherwise, our results suggest that no matter what level of subsidy is given to farmers, a high initial infestation level prevents any amount of spraying from improving the farm situation over a low initial infestation level.

Generalizing to all Hawaii coffee farms introduces limitations about the specificity of the model to individual farms. However, by incorporating individual farm-level parameters, the model calibrates to more accurately determine optimal decisions to specific farms. This data can then be used to provide real-time modeling throughout a growing season of a specific farm. Follow-up research will provide further details on incorporating individual farm-level parameters to address this concern with the model.

One of the main limitations of the model is the growth rate of CBB infestation is constant (i.e., 35%) for each period. In reality, the growth rate will vary, and is dependent upon many other factors such as weather conditions, elevation, externality effects from neighboring farms, and other crop management decisions. Current research on the biological reproduction of CBB will help to improve this formulation, but as it stands now no modeling of the growth rate is accurately reflective of Hawaii. To overcome this limitation, the model was calibrated using what is known from farm-level knowledge about the infestation level of CBB if a farmer chooses not to spray in all periods and chooses to spray [31,32]. Calibration from field tests determined 35% was the best fit overall.

Another limitation is pricing of the coffee crop is taken as exogenous and constant, whereas current industry practice is moving toward dynamic cherry pricing (i.e., pricing based on the infestation level as well as other measures of quality). Such pricing will have a major impact on grower incentives; a farmer with a lower level of infestation will receive a higher price for their cherry, so incorporating those effects on the total net benefit will ultimately improve the model.

To conclude, the decision tree model in this paper provides an understanding of when to spray or not spray by mapping decisions in order to maximize the net benefit over a coffee growing season. The model provides a visual representation of the decisions a farmer would make and allows for understanding sequential uncertainty in each period. These advantages allow for a general understanding of the parameters a farm will take into account and how each affects the total net benefit.

## Figures and Tables

**Table 1:** Farm Level Decisions.

	Divisor (Per)	Unit	Range	Average
Acres		Acres	0–3100	1.67
Projected Cherry	Acre	Lbs.	0–10,000	7500
Farm Labor	Hour	Dollars	0–30	15
Spray Labor	Acre	Hours	0–2	1
Harvest labor	Lbs.	Dollars	0–2	0.5
Pesticide	Acre	Quart	0–2	1
Pesticide Costs	Quart	Dollars	0–100	70.35
Water	Acre	Gallons	0–200	100
Water Cost	1k Gallon	Dollars	0–2	1
Surfactant	Acre	Ounces	0–100	45
Surfactant Costs	Quart	Dollars	0–20	8
Harvest Rate	Period	Percentage	0–100	25
Cherry Price	Lbs.	Dollars	0–2.5	2

**Table 2:** Infestation Rate Variables.

	Unit	Range	Average
Initial Infestation	%	0–10	1
Spray Effectiveness	%	0–100	50
Growth Rate	%	0–100	35

**Table 3:** Simulation Study 1 Analysis.

	Scenario 1 (Low Rate)	Scenario 2 (High Rate)	Difference
Initial infestation level	1%	6%	5%
Total NB (Optimal Path)	$15,478.09	$8716.95	$6761.14
Total NB (Spray)	$15,316.05	$8716.95	$6599.10

**Table 4:** Simulation Study 2 Analysis.

	Scenario 1 (No Subsidy)	Scenario 2 (w/Subsidy)	Difference
Pesticide Cost (per acre)	$70.35	$15.00	$55.35
Pesticide Cost (per month)	$117.48	$25.05	$92.43
Total NB (Optimal)	$15,478.09	$16,425.26	$947.17
Total NB (no spray final period)	$15,478.09	$16,402.43	$924.34

**Table 5:** Sensitivity Parameters.

	Min	Max	Base	Pessimistic Scenario	Optimistic Scenario
Cherry Price	$1.00	$2.00	$2.00	$1.50	$2.00
Spray Cost	$0	$100.00	$70.35	$70.35	$70.35
Infestation Level	1%	20%	1%	4%	1%
Spray Effectiveness	30%	80%	50%	40%	50%
CBB Growth Rate	0%	100%	35%	50%	25%

**Table 6:** Sensitivity of Net Benefit.

Initial Infestation	Net Benefit
0%	$16,635.87
1%	$15,246.59
2%	$13,857.30
5%	$9689.45
10%	$2743.04
12%	$1353.75
12.6%	$0.00
15%	−$3161.42
20%	−$9760.51

**Table 7:** Spray Effectiveness Sensitivity Analysis.

Spray Effectiveness	Net Benefit	Final Inf. Level	Net Harvests (Lbs.)
0%	$13,985.90	24%	10,124
25%	$14,245.52	17%	10,792
50%	$14,478.09	7.6%	11,767
75%	$16,257.25	5.5%	11,977
100%	$16,778.18	2.9%	12,238

**Table 8:** Pesticide Cost Sensitivity Analysis.

Pesticide Cost (per qt)	Optimal Net Benefit	Final Inf. Level	Net Harvest (Lbs.)
$0.00	$16,725.86	6.0%	11,865
$15.00	$16,425.26	6.0%	11,865
$20.40	$16,317.05	6.0%	11,865
$50.00	$15,817.93	7.6%	11,767
$70.35	$15,478.09	7.6%	11,767
$100.00	$14,982.93	7.6%	11,767
Difference			
70.35-0	$1247.77		
100.00-0	$1742.93		

**Table 9:** Breakeven on Initial Infestation.

**Spray Cost**	**Initial Infestation**
$0.00	13.67%
$25.00	13.29%
$50.00	12.91%
$75.00	12.53%
$100.00	12.15%
**Spray Effectiveness**	**Initial Infestation**
0%	3.47%
25%	6.38%
50%	12.61%
75%	27.27%
100%	66.41%

**Table 10:** Breakeven of Base Case.

Variable	Base	Breakeven
Pesticide Cost	$70.35	$834.62
Initial Infestation	1.00%	12.60%
Spray Effectiveness	50%	−59.69%
Growth Rate	35%	112%
Price	$2.00	$0.71

## Abbreviations

The following abbreviations are used in this manuscript:

CBB	Coffee Berry Borer
USDA	United States Department of Agriculture
IPM	Integrated Pest Management
